# American Public Health Association

**Published:** 1891-02

**Authors:** 


### AMERICAN PUBLIC HEALTH ASSOCIATION.

THE eighteenth annual meeting of this association was held at Charleston, S. C., on the 16th, 17th, 18th, and 19th of December, 1890, under the presidency of Dr. Henry B. Baker, of Lansing, Mich. There was an unusually large attendance at the meeting, nearly every state and territory in the Union being represented, as well as all the Canadian provinces. The Republic of Mexico was represented by Dr. Domingo Orvananos, of the Superior Board of Health of Mexico, and Prof. Jose L. Gomez, of the National School of Agriculture of Mexico. Prominent men from the medical corps of the U. S. Army and Navy were also in attendance. As an indication of the interest which is taken in this association, mention may be made that on the opening day about fifty new names were added to the membership list. Much of the success and pleasure of the meeting were due to the energetic and painstaking efforts of the chairman of the local committee of arrangements, Dr. Horlbeck, the efficient health officer of Charleston.

Made up, as this association is, of the foremost sanitarians of the country, a large number of practical and valuable papers were presented, embracing almost all topics falling within the domain of sanitary science and preventive medicine. The discussion which these various papers elicited was free and spirited and very generally indulged in. Papers relating to the subject of tuberculosis, and all things relating thereto, are at this time very important and fraught with great interest to mankind in general. The first paper relating to this matter was entitled The Federal District of Mexico as a Suitable Residence for Persons Predisposed to Tubercular Affections, and for the Relief of Pulmonary Consumption. This paper was written by Dr. Domingo Orvananos, of Mexico. It was translated into English and read by Dr. A. L. Gihon, U. S. N., as Dr. Orvananos does not speak English fluently. The paper was excellently written, and graphically presented the claims of that district. It showed careful study and research, and was fairly bristling with valuable statistics and information.

Dr. L. F. FLICK, of Philadelphia, next read a paper on the Prevention of Tuberculosis. This paper was largely historical and represented the results of a century's supervision in Italy under the influence of the preventive laws of the Kingdom of Naples, enacted in 1782. Dr. B. F. Wyman read a short paper on the Prevention of Phthisis. The question of the best climate for consumptives was largely entered into, and the paper incidentally, showed the great advantages of Aiken as a health resort, especially for those suffering from incipient pulmonary phthisis. Dr. Wyman has had fourteen years' experience at Aiken, and his views and opinions should possess considerable value.

Perhaps the most important discussion of the meeting followed the reading of these papers.

Dr. Gihon, of the U. S. Navy, said that something ought to be done to prevent the general expectoration of consumptives on cars, vessels and elsewhere. He hoped that a committee would be appointed to take the matter in hand. He remarked that the real object of the Association was to look after the prevention of the disease rather than its cure. Dr. Kemp, of Brooklyn, an expert bacteriologist, said that the bacilli of tuberculosis were very tenacious of life in their dry state. His own experiments and those of others had shown this, and he believed the disinfection of the sputa the keynote of the situation. Dr. Rohé, of Baltimore, said that if proper preventive measures were not taken at health resorts, it was useless for physicians to send their patients to these places. Prof. Johnson, of Chicago, advised the sending of tuberculous patients to cold and open regions where the bacilli do not flourish. Crowded cities were the worst possible places for consumptives. Dr. Beverely thought that the great mistake of consumptives was not to take up a permanent residence at some health resort. Dr. Ames, of the U. S. Navy, related how the people of Japan got rid of their sputa by carrying sheets of paper in their sleeves, which they used as handkerchiefs. These were afterward destroyed. Dr. Wyman, of the U. S. M. H. S., said something ought to be done to keep out of the country emigrants who have pulmonary troubles. After a protracted discussion Dr. Gihon offered the following resolution, which was unanimously adopted, "Resolved, That a standing committee of five be appointed by the president to formulate and distribute practical prophylactic rules and measures for the prevention of the spread of tuberculosis, especially looking to the protection of the healthy members of the community from tuberculous infection."

A very interesting paper was presented by Geo. T. Kemp, Ph. D., director of physiological and experimental therapeutics at the

Hoagland Laboratory, Brooklyn, N. Y., entitled Some Original Observations on the Microscopical, Spectroscopical and Chemical Experiments of Black Vomit as an Aid to Health Officers in Distinguishing between Yellow Fever and Malarial Fever. The report of Dr. Ashmun, of Cleveland, Ohio, chairman of the committee on the Cause and Prevention of Diphtheria, was an admirable document, teeming with valuable practical information relative to the prevention of this dreaded malady. It would be well if a copy of this valuable report could be placed in the hands of every board of health in the country. Protective Vaccination on Ocean Steamers was very ably presented by Dr. Montizambert, of the Province of Quebec. Dr. J. H. Raymond, of Brooklyn, N. Y., presented two very interesting papers which were illustrated with stereoptican views : one on The Treatment of Sewage by Precipitation and Saturation, the other on The Sanitary Improvement of Stagnant Lakes Near the Seashore. An original and interesting paper on Underground Water for Public Purposes was presented by Dr. P. H. Bryce, of Toronto, Ont. Dr. Jose L. Gomez gave the association some very elaborate and carefully prepared results of many original investigations in the hog-raising sections of Mexico in a paper entitled Swine Red Disease in Mexico. One of the most pleasing incidents of the meeting was a visit to the quarantine station at Fort Johnson, in Charleston harbor. For this purpose, the Secretary of the United States Treasury kindly placed at the disposal of the association the services of the United States revenue cutter Lot. M. Morrill. This trip enabled all who went to see the many objects of historical interest in and about the harbor, and to see, fully illustrated by the most sanitary and modern appliances, the methods employed at Charleston for fumigating and disinfecting infected ships, clothing, etc. On the return trip, a sumptuous collation was served on the steamer to all present. Another interesting trip was made, on invitation of the Emerson Car Ventilating Co., to the old plantation of Otranto. The cars used on this trip were ventilated by the Emerson appliance, and proved to be objects of great interest to many of the members. The old plantation house is now used as a country club, and here the members were treated to a royal repast of both solid and liquid refreshments. Among the social features of the occasion, perhaps the most important, was the public reception given to the members of the association at the Grand Opera House. Here the members were treated to some choice musical selections by the orchestra, after which Dr. Horlbeck introduced Dr. J. Somers Buist, of Charleston, who welcomed the Association to the city. He said Charleston was alive to the honor which had been done her in being selected as the meeting place of so distinguished a body. This was a body of scientific men, who, without hope of reward, were working to place truth before the world. They were striving to make system and order, displace ignorance and superstition, and to place before the public the need for health. In closing Dr. Buist paid a glowing tribute to Dr. Robert Koch, of Germany, and spoke eloquently of the great work he was now doing for science.

Dr. Buist was followed by President Baker, who delivered his annual address, which was full of valuable thought and suggestion. The object of the Association, he said, was implied in its name. It was devoted to the advancement of sanitary science and the promotion of public hygiene. Not only, he said, was the whole of this country interested in the work of this association, but so were also the provinces of Canada and the government of Mexico. Dr. Baker referred at some length to what the governments of Germany and Great Britain were doing for sanitation, and for public health generally. He referred in a fitting way to Koch and his great discovery. He discussed the bacilli of diphtheria, pneumonia, and other diseases incident to cold climes, and showed how by the work of health departments of various states and cities, many diseases had almost been wiped out. He argued that the United States should establish a health department of the interior, which was more needed than quarantine. The government should investigate typhoid and mountain fevers, and precautions should be taken against cholera. Measures should be taken to investigate infected localities, and the prevention of disease. He discussed at considerable length inflammatory diseases and their causes and prevention. He spoke at length, too, on the subject of government aid, and said that the constitution gave Congress the right to look after the general welfare of the people, and it could not be better done than by providing for the investigation and prevention of diseases. Political questions, such as free trade and protection, were of small consequence when compared to governmental protection to life and health. Rev. Dr. Vedder and Mr. P. K. Bryan also made eloquent addresses at this meeting. While in Charleston, many members of the association, on invitation, availed themselves of the courtesies of the Charleston Club. An invitation from Dr.

Wyman was extended to the association to visit Aiken, S. C., which is now enjoying great popularity as a health resort, and after the final adjournment many of the members went to Aiken on a special train provided for the purpose, where they spent a couple of days very pleasantly. Buffalo was represented at the meeting by Health Physician W. D. Greene, ex-Health Physician Edward Clark, City Chemist F. P. Vandenbergh, and Dr. A. R. Wright. Dr. Montizambert, of Quebec, was elected president, and Dr. Thos. F. Wood, of Wilmington, N. C., first vice-president. The secretary and treasurer, and other officers were all reëlected. Buffalo's representatives fared pretty well so far as the appointment on committees was concerned. Dr. Greene was placed on the committee on the Cause and Prevention of Diphtheria, Dr. Vandenbergh on the committee on the Pollution of Water Supply, and Dr. Clark was elected a member of the executive committee, and was placed also on the committee on the disposal of Garbage and Refuse of cities. It was voted to hold the next meeting of the association at Kansas City, Mo., in November, 1891. E. C.

